# Syk facilitates phagosome-lysosome fusion by regulating actin-remodeling in complement-mediated phagocytosis

**DOI:** 10.1038/s41598-020-79156-7

**Published:** 2020-12-16

**Authors:** Hiroyuki Tabata, Hiroyuki Morita, Hiroaki Kaji, Kaoru Tohyama, Yumi Tohyama

**Affiliations:** 1 Division of Biochemistry, Faculty of Pharmaceutical Sciences, Himeji Dokkyo University, 7-2-1 Kami-ohno, Himeji, Hyogo 670-8524 Japan; 2 Department of Laboratory Medicine, Kawasaki Medical School, Okayama, 701-0192 Japan

**Keywords:** Immunology, Microbiology

## Abstract

Effective phagocytosis is crucial for host defense against pathogens. Macrophages entrap pathogens into a phagosome and subsequently acidic lysosomes fuse to the phagosome. Previous studies showed the pivotal role of actin-remodeling mediated by phosphoinositide-related signaling in phagosome formation, but the mechanisms of phagosome-lysosome fusion remain unexplored. Here we show that in complement-mediated phagocytosis, phagosome-lysosome fusion requires the disappearance of F-actin structure surrounding the phagosome and a tyrosine kinase Syk plays a key role in this process. Using macrophage-like differentiated HL60 and Syk-knockout (Syk-KO) HL60 cells, we found that Syk-KO cells showed insufficient phagosome acidification caused by impaired fusion with lysosomes and permitted the survival of *Candida albicans* in complement-mediated phagocytosis. Phagosome tracking analysis showed that during phagosome internalization process, F-actin surrounding phagosomes disappeared in both parental and Syk-KO cells but this structure was reconstructed immediately only in Syk-KO cells. In addition, F-actin-stabilizing agent induced a similar impairment of phagosome-lysosome fusion. Collectively, Syk-derived signaling facilitates phagosome-lysosome fusion by regulating actin-remodeling.

## Introduction

Efficient phagocytosis of pathogens is crucial for host defense mechanisms, and macrophages act as professional phagocytes in concert with neutrophils and dendritic cells. Macrophages express a variety of phagocytic receptors, such as the Fc receptor, C-type lectin receptors, and complement receptor 3 (CR3, CD11b/CD18, integrin αMβ2). Phagocytosis is triggered by an association between ligands on the surface of pathogens and receptors on the membrane of phagocytes. Complement-mediated phagocytosis is initiated by binding of complement component C3bi to CR3. Complement system is comprised of cascade reactions that converge on the formation of complement component C3b. C3b and its inactive fragment C3bi are recognized by CR3 on phagocytes. Particularly, C3bi is a strongest inducer of complement-mediated phagocytosis.

Syk is a non-receptor protein tyrosine kinase expressed in a wide range of hematopoietic cells, plays essential roles in both innate and adaptive immune responses, and further acts as a regulator of inflammatory diseases^1–5^. Syk mediates signaling pathways evoked by various types of receptors including classical immunoreceptors, integrins and C-type lectins^6–11^. As for integrin-mediated signaling, important roles of Syk related to actin-remodeling were reported in various cells such as platelets, megakaryocytes, osteoclasts and phagocytic cells including macrophages and neutorphils^10,12–16^. About phagocytosis, we previously reported a critical role of Syk in complement-mediated phagocytosis^10^.

Phagocytosis requires dynamic and coordinated reconstruction of the membrane and underlying cytoskeleton. Pathogens are entrapped into a phagosome followed by internalization and subsequently acidic lysosomes fuse to the phagosome^17^. Previous studies showed pivotal roles of phosphoinositide-mediated signaling and actin-remodeling in phagosome formation process but the mechanisms of phagosome-lysosome fusion remain unexplored^18–20^.

In the current study, we focus on the mechanisms that regulate phagosome-lysosome fusion in complement-mediated phagocytosis. Using Syk-knock out (KO) cells and an F-actin-stabilizing agent, we demonstrated that phagosome-lysosome fusion requires the disappearance of F-actin structure surrounding phagosomes and Syk-derived signaling accelerates this fusion by regulating actin-remodeling.

## Results

### Syk-KO macrophages permit escape of *C. albicans* from the phagosome

**Figure 1:**
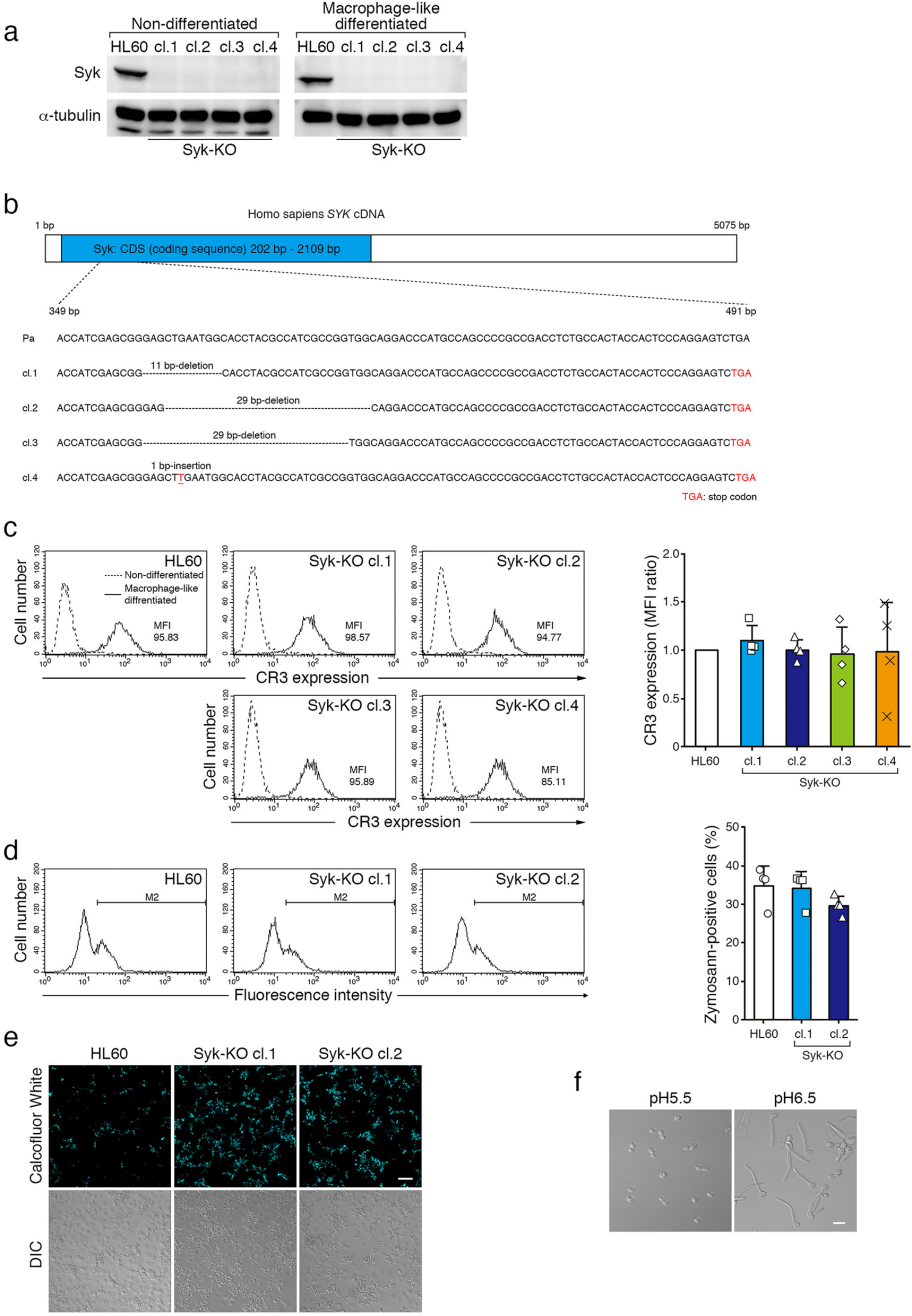
Complement-mediated phagocytosis of macrophage-like differentiated Syk-KO HL60 cells. (**a**) Immunoblotting analysis of Syk in parental HL60 and 4 clones of Syk-KO HL60 cells (left; non-differentiated, right; macrophage-like differentiated). The original images of the immunoblotting analysis are presented in Supplementary information. (**b**) Sequence comparison of *syk* gene between parental and Syk-KO HL60 cell lines. (**c**) Representative histograms of cell surface expression of CR3 in macrophage-like differentiated parental or Syk-KO HL60 cells. (**d**) Binding of complement-opsonized zymosan particles (Texas Red-labeled) to CR3 expressed in the macrophage-like differentiated parental and Syk-KO HL60 cells. In (**c**, **d**), left panel shows representative histograms assessed by flow cytometry. Right panel shows quantification of the ratio of MFI (**c**) and the percentage of zymosan-positive cells (**d**), respectively. Data show the means ± SD derived from four independent experiments. (**e**) *C. albicans* stained with Calcofluor White that was phagocytosed by macrophage-like differentiated parental or Syk-KO HL60 cells for 15 min, followed by wash and incubation for another 8 h. (**f**) *C. albicans* incubated for 3 h in the 50 mM citrate (pH 5.5) or phosphate (pH 6.5) buffer containing 10% FCS. In (**e**) and (**f**), DIC indicates differential interference images.

To reveal the role of Syk in complement-mediated phagocytosis, particularly phagosome-lysosome fusion, we established various Syk-knockout (Syk-KO) cell lines by using CRISPR/Cas9 system in HL60 cells. Both CRISPR/Cas9 protein and the specific guide RNA targeting the exon of human *syk* gene were co-transfected into parental HL60 cells, and Syk-KO cell clones were selected by immunoblotting analysis (Fig. 1a, Fig. S1a, b). Sequence analysis confirmed that 4 types of Syk-KO cell clones, having different insertion-deletion mutations were established (Fig. 1b). Flow cytometric analysis revealed no apparent difference in cell surface expression of complement receptor 3 (CR3) between parental and Syk-KO HL60 cells (Fig. 1c).

Using these parental and mutant HL60 cells, we performed phagocytosis assay as previously described^10^. Macrophage-like differentiated cells were incubated with complement-opsonized fluorescence-labeled zymosan particles for 15 min, washed with ice-cold PBS to remove un-incorporated particles and these cells were further incubated. Flow cytometric analysis showed that Syk-KO has no apparent effect on binding of CR3 to opsonized zymosan particles (Fig. 1d).

Next, to examine the effect of Syk-KO on microbicidal activity of phagocytosis, we performed phagocytosis assay using complement-opsonized *Candida albicans* (*C. albicans*). Eight hours after initiation of phagocytosis, we found that larger number of *C. albicans* developed hyphae after phagocytosis by Syk-KO cells than by parental cells (Fig. 1e). These results suggest that intra-phagosomal milieu of Syk-KO is suitable for hyphal elongation of *C. albicans* and this pathogen escaped from Syk-KO phagosomes more readily than from phagosomes of parental HL60 cells. In general, phagosome is gradually acidified through the fusion with lysosomes during phagocytic process of macrophages. To determine whether hyphal elongation is affected by intra-phagosomal pH, we incubated *C. albicans* in the medium adjusted at pH 5.5 or pH 6.5. Hyphal elongation was evidently suppressed in the medium at pH 5.5 (Fig. 1f). In consequence, we concluded that phagosome acidification is a critical process of inhibition for hyphal elongation and this process is impaired in macrophage-like differentiated Syk-KO cells.

### Syk facilitates acidification of phagosomes

**Figure 2:**
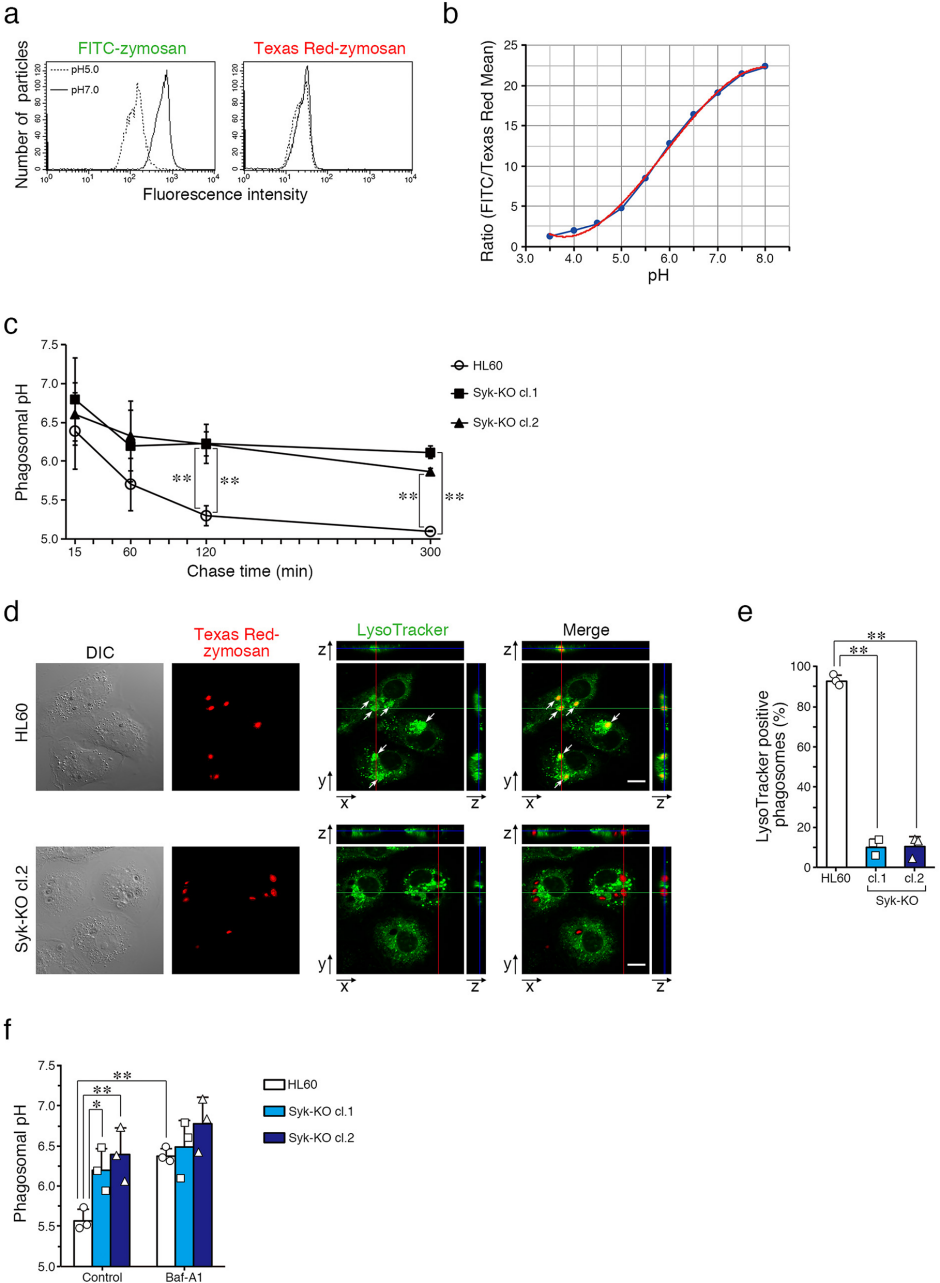
Syk-KO attenuates phagosome acidification. (**a**) Representative histograms of fluorescence intensity of FITC- labeled or Texas Red-labeled zymosan particles in pH 7.0 phosphate buffer or pH 5.0 citrate buffer. (**b**) Ratio of fluorescence intensity of two types of fluorescence-labeled zymosan particles (FITC-/ Texas Red-) in various pH conditions among pH 3.5–8. (**c**) Time-dependent change of phagosomal pH in macrophage-like differentiated parental or Syk-KO HL60 cells after the initiation of complement-mediated phagocytosis using two types of fluorescence-labeled zymosan particles. (**d**) Representative images of macrophage-like differentiated parental and Syk-KO HL60 cells after the initiation of phagocytosis using complement-opsonized Texas Red-labeled zymosan particles in the presence of LysoTracker Green, followed by wash and incubation for 2 h, as quantified in (**e**). Arrows indicate LysoTracker-postive phagosomes. Scale bars show 10 μm. (**e**) Ratio of LysoTracker-positive phagosomes of the indicated cells. More than 100 phagosomes per single clone were analyzed. (**f**) Phagosomal pH of macrophage-like differentiated parental and Syk-KO HL60 cells after the initiation of phagocytosis using two types of fluorescence-labeled zymosan particles, followed by incubation for 90 min and further incubated for 30 min in the presence or absence of bafilomycin A1. In (**c**, **e**, **f**), data show the means ± SD derived from three independent experiments. *p* values were calculated using a two-tailed unpaired Student’s *t*-test. ** < 0.01; * < 0.05.

To confirm the effects of Syk-KO on phagosome acidification, we used two types of fluorescence-labeled zymosan particles, one was fluorescein isothiocyanate (FITC)-labeled particles whose fluorescence intensity is reduced with the decrease of pH, and the other was Texas Red-labeled particles whose intensity is conserved independent of pH change (Fig. 2a). Using flow cytometry, we analyzed the ratio of fluorescence intensity of two types of fluorescence-labeled zymosan particles in various pH conditions among pH 3.5–8.0 and determined the standard curve for estimating phagosome pH (Fig. 2b). Then, we quantified the phagosome pH of macrophage-like differentiated HL60 and Syk-KO cells by performing phagocytosis assay using both FITC- and Texas Red-labeled zymosan particles. Flow cytometric analysis showed that pH value of phagosome in parental HL60 cells decreased gradually in a time-dependent fashion and reached below pH 5.5 at 2 h after incubation, but the pH value of phagosomes in Syk-KO cells remained above pH 6. After 5 h, the pH value of phagosomes of parental cells showed pH 5 but that of Syk-KO was around pH 6 (Fig. 2c), indicating the acidification defect of Syk-KO phagosomes.

Next, we examined the phagosome acidification microscopically using acidic organelle selective fluorescent probe, LysoTracker Green. At 2 h after initiation of phagocytosis, most phagosomes of parental HL60 cells were fluorescence-positive but few phagosomes of Syk-KO cells were positive (Fig. 2d,e). This result also indicates that Syk-KO inhibits acidification of phagosome. These results showed that Syk plays a significant role in facilitating phagosome acidification in complement-mediated phagocytosis.

To ascertain if the acidification of phagosomes is brought about by the function of proton pump, we treated bafilomycin A1, a vacuolar-type H^+^-ATPase (V-ATPase)-specific inhibitor. Treatment with bafilomycin A1 completely abrogated phagosome acidification of both parental and Syk-KO cells and phagosomal pH was retained around pH 6.5 (Fig. 2f).

These data suggest that phagosome acidification initiated by complement-mediated phagocytosis is due to the function of lysosome-derived proton pump. How Syk facilitates the acidification of the phagosomes? We examined the two possibilities: Syk up-regulates the function of the proton pump or Syk increases the amount of the pump that exists in the phagosome.

### Syk facilitates fusion of phagosomes with lysosomes

**Figure 3:**
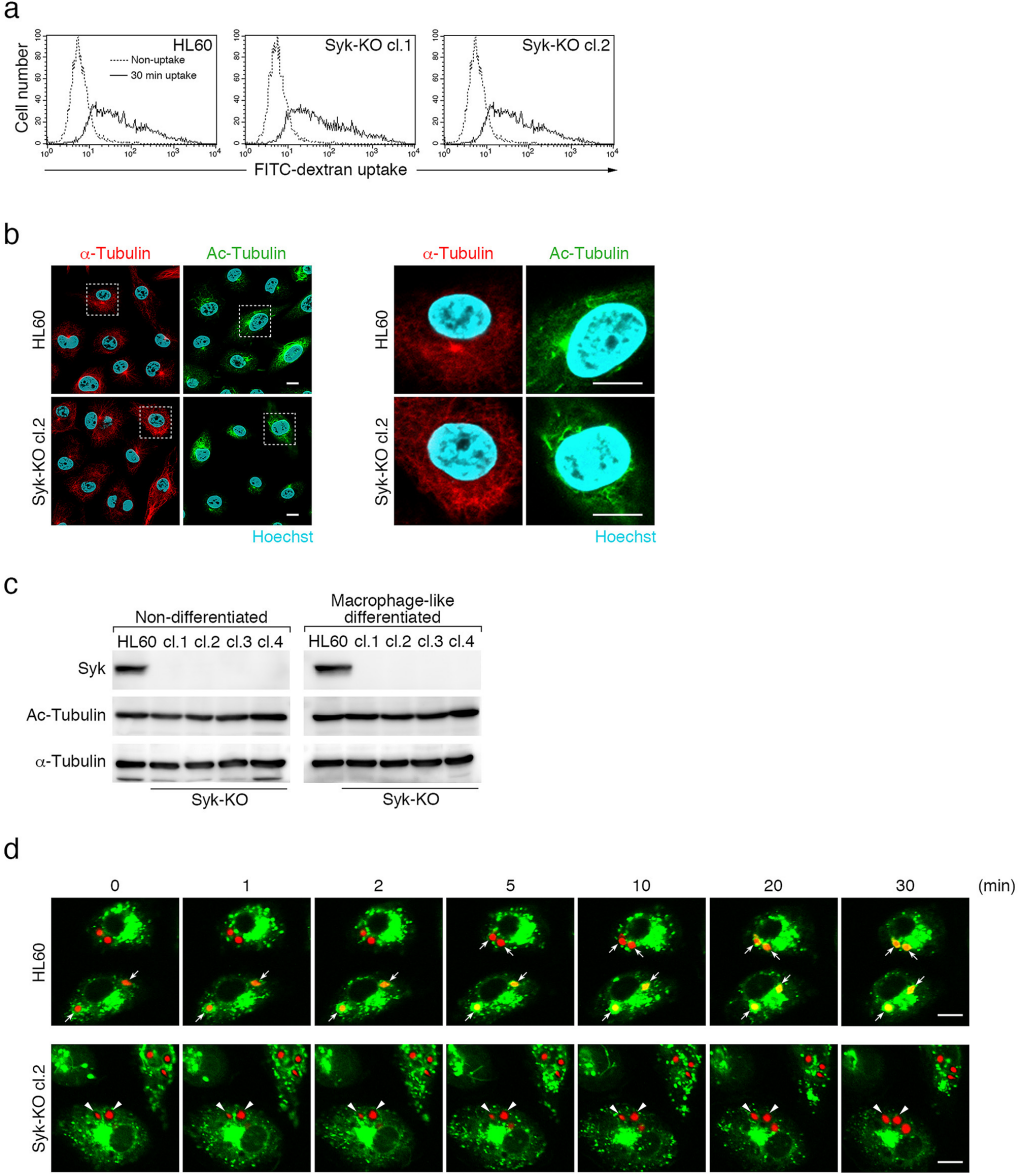
Syk-KO inhibits phagosome-lysosome fusion. (**a**) Representative histograms of fluorescence intensity of macrophage-like differentiated parental and Syk-KO HL60 cells after incubation for 30 min in the presence of FITC-labeled dextran. (**b**) Representative images of immunofluorescence staining of macrophage-like differentiated parental and Syk-KO HL60 cells using antibodies against α-tubulin (red) or acetylated tubulin (green). Nuclei are stained blue with Hoechst33342. Scale bars show 10 μm. (**c**) Immunoblotting analysis of α-tubulin or acetylated tubulin in parental and Syk-KO HL60 cells. The original images of the immunoblotting analysis are presented in Supplementary information. (**d**) Time lapse imaging of phagocytosis of macrophage-like differentiated parental and Syk-KO HL60 cells in the presence of LysoTracker Green, after the incubation of complement-opsonized Texas Red zymosan particles for 15 min, followed by wash and incubation for the indicated times. Arrows show LysoTracker-positive phagosomes and arrowheads show LysoTracker-negative phagosomes. Scale bars show 10 μm.

To examine whether Syk-KO affects the function of proton pump in lysosomes, we treated macrophage-like differentiated cells with pH-sensitive fluorescent probe, FITC-labeled dextran, which is endocytosed and accumulated in the lysosomal compartment. Flow cytometric analysis showed no apparent difference in fluorescence intensity between parental and Syk-KO HL60 cells (Fig. 3a). These results suggest that Syk-KO cells have functional V-ATPase in lysosomes and the defect of phagosome acidification in Syk-KO cells is not due to dysfunction of the proton pump, V-ATPase in lysosomes. Then, we presumed that defect of phagosome acidification in Syk-KO cells is caused by impaired fusion of phagosome with lysosome. We generated two hypotheses as follows. One was that lysosomes failed to reach phagosomes by the defect of intracellular transport and the other was that the final stage of phagosome-lysosome fusion is directly inhibited by an unknown molecular mechanism.

To determine whether lysosome transport into phagosomes is impaired in Syk-KO cells, we investigated the acetylation of α-tubulin at lysine 40 (K40) focusing on intracellular distribution and the amount of modification, because trafficking of lysosomes is reported to be affected by this post-translational modification^21^. Both microscopic analysis and immunoblotting analysis showed little difference in the distribution and in the amount of acetylation of α-tubulin (K40), between Syk-KO and parental HL60 cells (Fig. 3b,c, Fig. S2a–c).

Next, we tracked phagosome dynamics paying attention to the interaction with lysosomes. Imaging analysis revealed that in parental HL60 cells lysosomes fused to phagosomes sequentially, whereas in Syk-KO cells, lysosomes approached close to phagosomes but failed to fuse to phagosomes (Fig. 3d and Supplementary Video 1, 2).

These results suggest that the lysosomal transport toward phagosomes is intact but subsequent lysosome-phagosome fusion is impaired in Syk-KO cells.

### Syk promotes phagosome-lysosome fusion by interrupting the formation of F-actin structure around phagosomes

**Figure 4:**
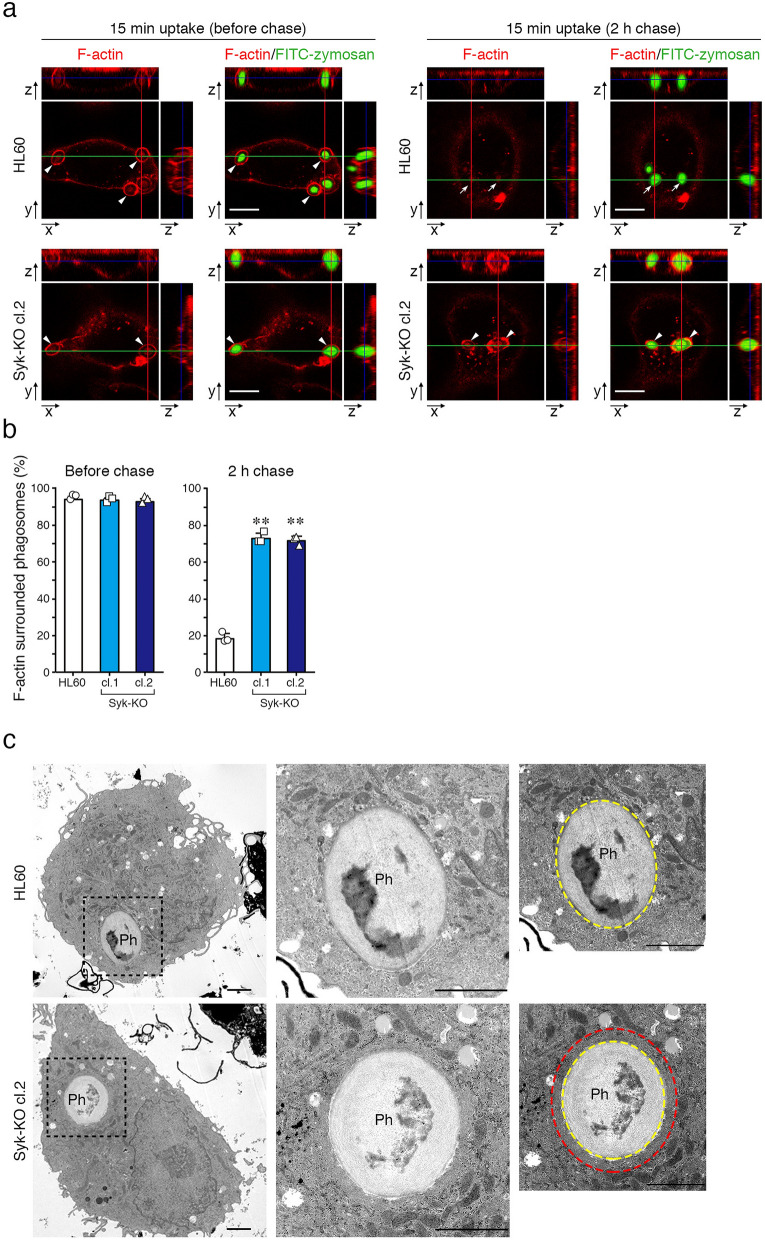
Syk-KO inhibits the disappearance of F-actin around phagosomes. (**a**) Representative images of F-actin (Alexa Fluor 594-labeled phalloidin) during phagocytosis of macrophage-like differentiated parental and Syk-KO HL60 cells after incubation with complement-opsonized FITC-zymosan particles for 15 min, followed by incubation for indicated time intervals, as quantified in (**b**). Arrows show the phagosomes that are not surrounded by F-actin and arrowheads show the phagosomes surrounded by F-actin. Scale bars show 10 μm. (**b**) Quantification of the percentage of the phagosomes surrounded by F-actin in macrophage-like differentiated parental and Syk-KO HL60 cells during phagocytic process. More than 100 phagosomes per single clone were analyzed. Data show the means ± SD derived from three independent experiments. *p* values were calculated using a two-tailed unpaired Student’s *t*-test. ** < 0.01; * < 0.05. (**c**) Representative electron micrographs of macrophage-like differentiated parental and Syk-KO HL60 cells after incubation with complement-opsonized FITC-zymosan particles for 15 min, followed by incubation for additional 2 h. Phagosomes (Ph) enclosed in dash squares are enlarged in the middle and right panels. Dashed circles indicate surface of phagosome surrounded by F-actin (red), and surface of zymosan particles (yellow), respectively. Scale bars show 2 μm.

We previously reported that Syk accelerates the engulfment of pathogen by participation in actin dynamics in complement-mediated phagocytosis^10^. Then, we speculated that Syk also contributes to actin dynamics in the regulation of phagosome-lysosome fusion. To test the hypothesis, we analyzed the distribution of F-actin surrounding phagosomes using fluorescence-labeled phalloidin, an F-actin specific indicator. At 15 min after addition of complement-opsonized zymosan particles, in both parental and Syk-KO HL60 cells, almost all phagosomes were surrounded by F-actin but they were not deeply internalized into the cell. At 2 h after further incubation, most of phagosomes were internalized and became free from F-actin in HL60 cells but in Syk-KO cells about 70% of the phagosomes still remained surrounded by F-actin (Fig. 4a,b). To confirm the effect of Syk-KO on F-actin around phagosomes, we utilized *Staphylococcus aureus* (*S. aureus)* as another phagocytic target. A similar difference of F-actin surrounding phagosomes was observed between HL60 cells and Syk-KO cells (Fig. S3a, b). Transmission electron microscopy (TEM) imaging also confirmed that phagosomes were surrounded by thick F-actin-like structure only in Syk-KO cells (Fig. 4c). These results suggest that Syk is committed to the F-actin dynamics around phagosomes in relation to internalization process.

**Figure 5:**
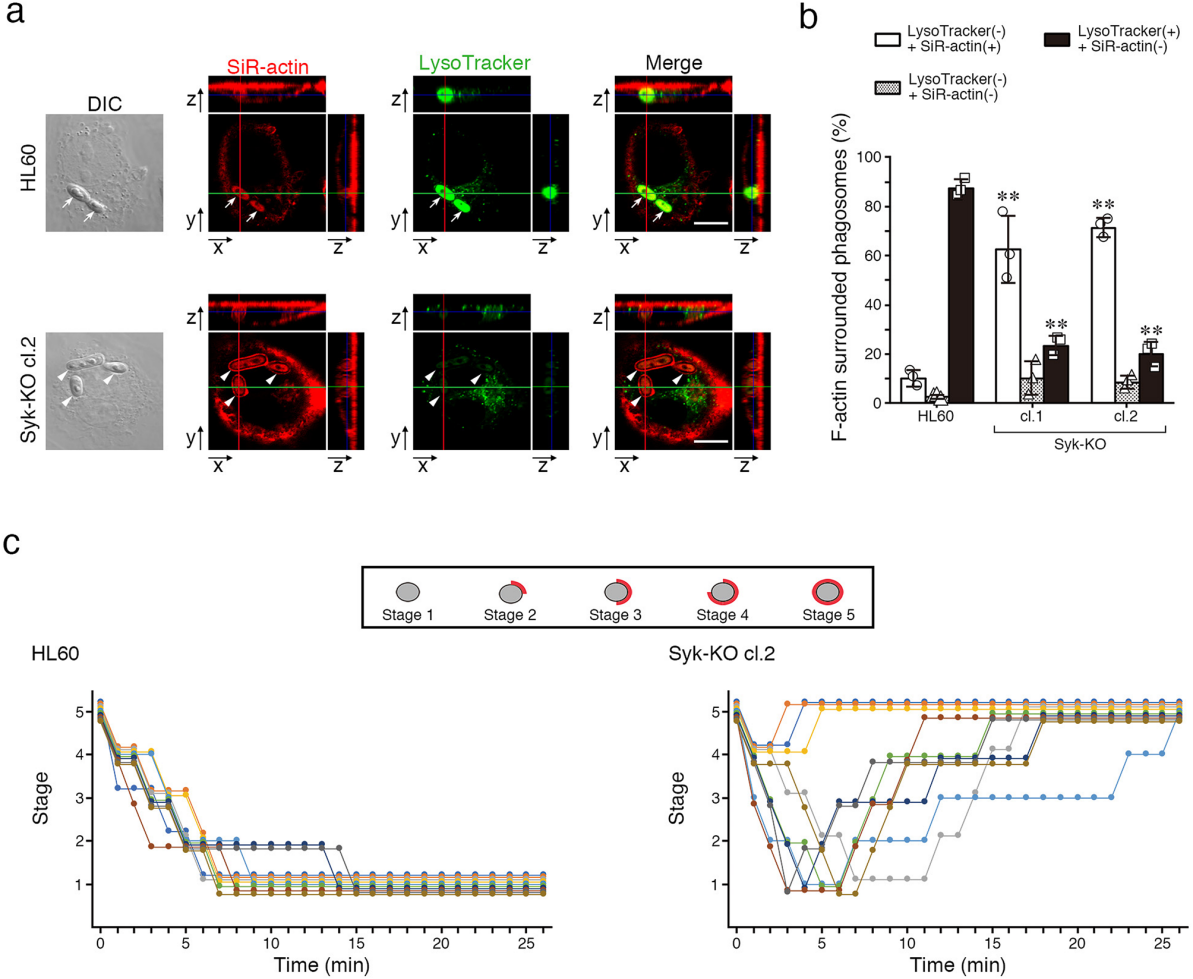

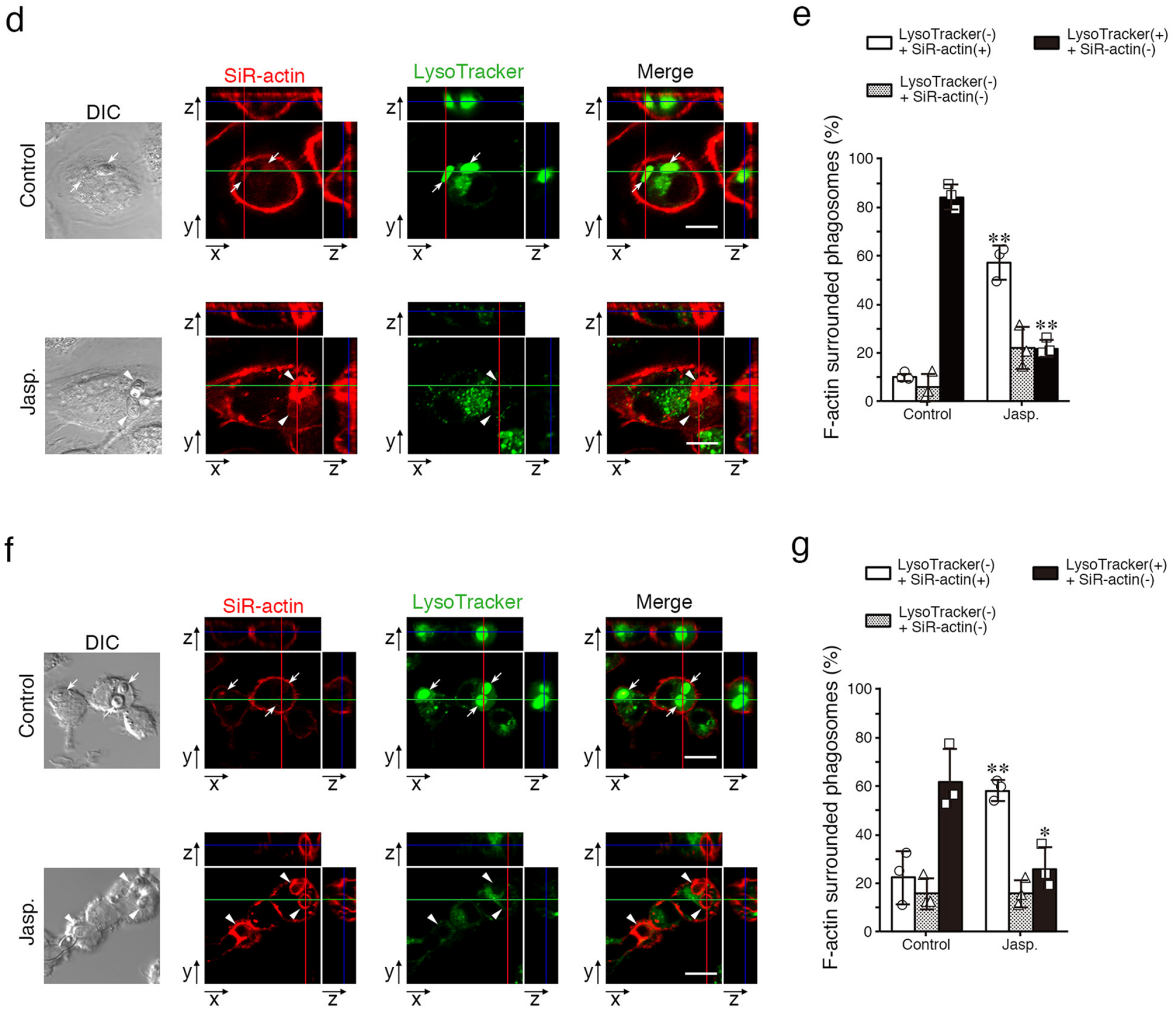
Syk-KO inhibits phagosome-lysosome fusion by re-construction of F-actin around phagosomes. (**a**) Representative live imaging of phagocytosis of macrophage-like differentiated parental and Syk-KO HL60 cells in the presence of LysoTracker Green and SiR-actin, after incubation with complement-opsonized-heat killed *C. albicans* for 15 min, followed by incubation for additional 2 h, as quantified in (**b**). Arrows show the phagosomes that are not surrounded by SiR-actin and arrowheads show the phagosomes surrounded by SiR-actin. Scale bars shows 10 μm. (**b**) Percentage of phagosomes classified into 3 groups focusing on the existence of LysoTracker and SiR-actin around phagosomes in macrophage-like differentiated parental and Syk-KO HL60 cells: LysoTracker-negative and surrounded by SiR-actin(+) (white); LysoTracker-negative and not surrounded by SiR-actin(−) (mesh); LysoTracker-positive and not surrounded by SiR-actin(−) (black). More than 100 phagosomes per single clone were analyzed. (**c**) Tracking analysis of F-actin status around individual phagosomes by the minute. Ten phagosomes were tracked from sequential live cell images in both cells just after formation of the phagosomes fully surrounded by SiR-actin. Live cell images of each phagosome were classified into 5 stages by the degree of F-actin surrounding phagosome (Stage1-5). Phagosomes are fully surrounded by F-actin (Stage 5); semi to fully surrounded (Stage 4); quarter to semi surrounded (Stage 3); non to quarter surrounded (Stage 2); non-surrounded (Stage 1). F-actin (red) and phagosomes (gray). (**d**, **f**) Representative live imaging of phagocytosis of parental HL60 cells (**d**) or primary human monocyte-derived macrophages (**f**) in the presence of LysoTracker Green and SiR-actin, after incubation with complement-opsonized-heat killed *C. albicans* for 15 min, followed by incubation for additional 1 h in the presence or absence of jasplakinolide, as quantified in (**e**) or **(g**). Arrows show the phagosomes that are not surrounded by SiR-actin and arrowheads show the phagosomes surrounded by SiR-actin. Scale bars shows 10 μm. In (**e**, **g**), percentage of phagosomes classified into 3 groups focusing on the existence of LysoTracker and SiR-actin around phagosomes was indicated as shown in (**b)**. More than 60 phagosomes were analyzed. In (**b**, **e**, **g**), data show the means ± SD derived from three independent experiments. Results were compared using one-way ANOVA. **p < 0.01; *p < 0.05.

To further analyze F-actin dynamics in detail, we tracked phagosomes containing heat-killed *C. albicans* in the presence of SiR-actin, a fluorescent probe for live-cell imaging of F-actin and LysoTracker Green. As a result, just after phagosome internalization, F-actin surrounding phagosomes clearly disappeared in parental HL60 cells and such phagosomes became free from F-actin. At 2 h after the initiation of phagocytosis, fluorescence-labeled SiR-actin surrounding phagosomes attenuated and instead the fluorescence of LysoTracker Green was detected in the phagosomes of parental HL60 cells (Fig. 5a,b and Supplementary Video 3). In contrast, in Syk-KO HL60 cells, F-actin surrounding phagosomes was once decreased during internalization process, but immediately after the process, F-actin newly appeared and surrounded the phagosomes again and fluorescence of LysoTracker Green was invisible (Fig. 5a,b and Supplementary Video 4). Tracking analysis of the individual phagosomes demonstrated that F-actin surrounding phagosomes disappeared and remained invisible in parental HL60 cells, whereas in Syk-KO cells, F-actin structure was eventually re-constructed around phagosomes after internalization even though the extent and kinetics of F-actin reduction/re-acquisition varied among phagosomes (Fig. 5c, and Supplementary Video 3, 4).

To confirm that the disappearance of F-actin is essential for phagosome-lysosome fusion, we treated parental HL60 cells with jasplakinolide, a stabilizing agent of F-actin and performed complement-mediated phagocytosis assay. To avoid any effect of jasplakinolide on the early stage of phagocytosis, we added the drug at 15 min after the initiation of phagocytosis. Fluorescence imaging revealed that treatment with jasplakinolide retained F-actin structure and inhibited fusion with acidic lysosomes (Fig. 5d,e). In addition, we treated human peripheral blood-derived monocytes with jasplakinolide, performed phagocytosis assay and confirmed almost the same effect on F-actin structure of monocytes as the case of HL60 (Fig. 5f,g).

These results suggest that F-actin structure surrounding phagosome interrupted the fusion with lysosomes. In other words, disappearance of F-actin structure surrounding phagosome is essential for the fusion with lysosomes and Syk plays a critical role in this process.

**Figure 6:**
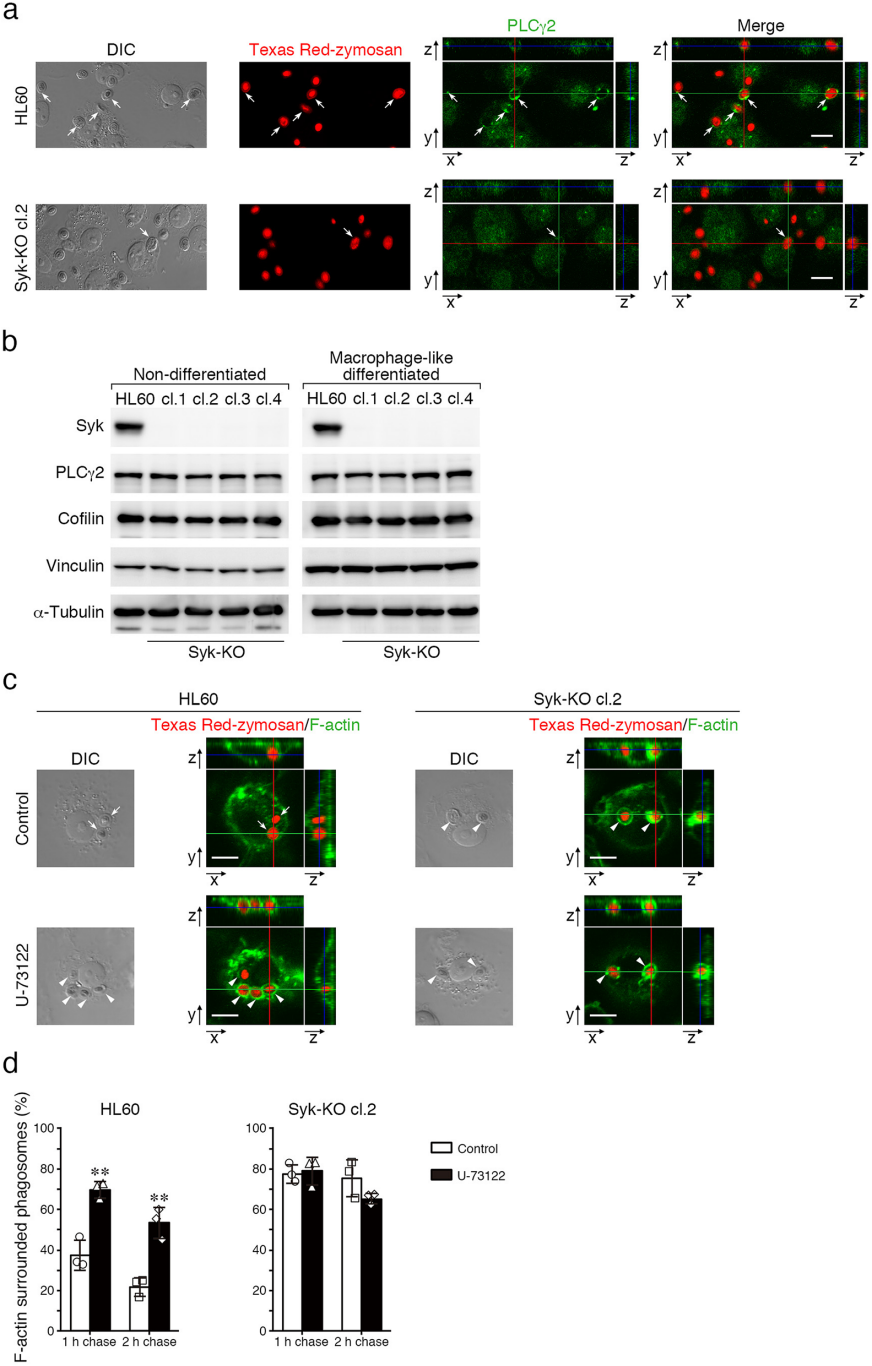
Effects of Syk-KO on the expression of actin-binding proteins. (**a**) Representative images of immunofluorescence staining of macrophage-like differentiated parental and Syk-KO HL60 cells using antibodies against PLCγ2 (green) after incubation with complement-opsonized Texas Red-labeled zymosan particles for 15 min. Scale bars show 10 μm. (**b**) Immunoblotting of Syk, PLCγ2, Cofilin, Vinculin, and α-tubulin in parental and Syk-KO HL60 cells (left; non-differentiated, right; macrophage-like differentiated). The original images of the immunoblotting analysis are presented in Supplementary information. (**c**) Representative images of F-actin (Alexa Fluor 488-labeled phalloidin) during phagocytosis of macrophage-like differentiated parental and Syk-KO HL60 cells after incubation with complement-opsonized Texas Red-zymosan particles for 15 min, followed by incubation for additional 1 h in the presence or absence of U-73122. Arrows show the phagosomes that are not surrounded by F-actin and arrowheads show the phagosomes surrounded by F-actin. Scale bars shows 10 μm. (**d**) Quantification of the percentage of the phagosomes surrounded by F-actin in macrophage-like differentiated parental and Syk-KO HL60 cells during phagocytic process in the presence or absence of U-73122 (1 h and 2 h). More than 150 phagosomes per single condition were analyzed. Data show the means ± SD derived from three independent experiments. *p* values were calculated using a two-tailed unpaired Student’s *t*-test. ** < 0.01.

What kind of signaling pathways work at the downstream of Syk in actin remodeling? We analyzed the distribution of phospholipase Cγ2 (PLCγ2) during phagosome formation process, because this protein has been known to act as a regulator of F-actin via binding of actin-binding proteins. PLCγ2 accumulated around phagosomes in parental HL60 cells but in Syk-KO cells the accumulation reduced to about a half (accumulation-positive phagosomes: 58% in HL60 cells, 33% in Syk-KO HL60 cells), although the expression of PLCγ2 was unchanged between parental and Syk-KO HL60 cells by immunoblotting analysis (Fig. 6a,b). Similarly, the expression of actin-regulating proteins, cofilin and vinculin was also unchanged between these cells (Fig. 6b, Fig. S4a–d). Next, to examine the role of phospholipase C activity in F-actin structure surrounding phagosomes, we treated parental and Syk-KO HL60 cells with a phospholipase C inhibitor, U-73122 and performed complement-mediated phagocytosis assay. To avoid any effect of U-73122 on the early stage of phagocytosis, we added this inhibitor at 15 min after the initiation of phagocytosis. Fluorescence imaging revealed that the ratio of phagosomes surrounded by F-actin was increased in parental HL60 cells but not in Syk-KO cells by the treatment with this inhibitor (Fig. 6c,d). Furthermore, we showed that functional Syk (phosphorylated at Tyr525/526) and PLCγ2 were located around both F-actin-surrounded and F-actin-free phagosomes (Fig. S5). These results suggested that the PLCγ2 is one of the possible candidates which regulate F-actin dynamics at the downstream of Syk and determine lysosome fusion.

## Discussion

In the present study, we demonstrate that in complement-mediated phagocytosis, phagosome-lysosome fusion requires disappearance of F-actin structure surrounding phagosomes to permit the contact of lysosomes to phagosomes and Syk plays a critical role in facilitating the fusion by regulating actin-remodeling.

We firstly found that larger number of *C. albicans* phagocytosed by Syk-KO HL60 cells survived than those phagocytosed by parental HL60 cells and found that phagosome acidification is impaired in Syk-KO cells (Figs. 1e,f, 2). Next, we showed that the defect of phagosome acidification in Syk-KO cells is caused by impaired phagosome**-**lysosome fusion, neither by dysfunction of the proton pump in lysosomes nor by the lysosomal transport toward phagosomes (Fig. 3).

Why Syk-KO leads to impaired phagosome**-**lysosome fusion? We focused on the effect of Syk on actin-remodeling and found that phagosomes in Syk-KO cells were surrounded by thick F-actin structure after internalization into the cells (Fig. 4). Tracking analysis of the individual phagosomes using a F-actin fluorescent probe for live-cell imaging clearly showed that F-actin surrounding phagosomes disappeared and remained invisible in parental HL60 cells, but in Syk-KO cells F-actin structure was re-constructed around phagosomes after internalization (Fig. 5c, and Supplementary Video 3, 4). In addition, treatment with F-actin stabilizing agent, jasplakinolide brought a similar fusion impairment in both parental HL60 cells and human peripheral blood-derived monocytes (Fig. 5d–g). Collectively, disappearance of F-actin structure surrounding phagosomes is a critical process in the phagosome**-**lysosome fusion and Syk plays a key role in this process.

**Figure 7:**
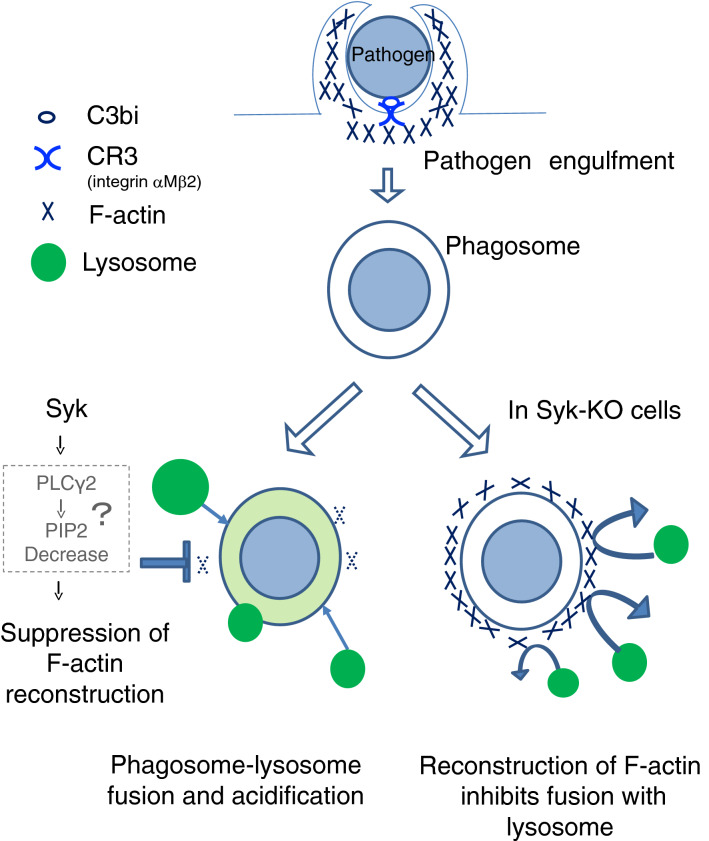
A scheme of the hypothesis that Syk-derived signaling facilitates phagosome-lysosome fusion by regulating actin-remodeling. Dashed square shows the hypothetical signaling derived from Syk leading to suppression of F-actin reconstruction.

What kind of signaling pathways work at the downstream of Syk in actin-remodeling? As a candidate molecule, we pay attention to PLCγ2, because it is known to regulate the amount of PtdIns(4,5)P_2_ and act as a regulator of F-actin in phagocytosis^20^. Previously, Syk-PLCγ2 signaling has been reported in immune and hematopoietic cells, B cell receptor-signaling, integrin signaling in neutrophils and megakaryocytes^22–25^. Our results indicated that at 15 min after initiation of phagocytosis, accumulation of PLCγ2 around phagosomes was reduced in Syk-KO cells (Fig. 6a). Furthermore, treatment with a phospholipase C inhibitor, U-73122 increased the ratio of phagosomes surrounded by F-actin only in parental HL60 cells but not in Syk-KO cells (Fig. 6c,d). Considering both these previous data and our results, the hypothesis might be raised as follows: during the early-stage of complement-mediated phagocytosis, PtdIns(4,5)P_2_ produced by PtdIns(4)P-5-kinases accelerates actin polymerization and F-actin structure surrounds phagosomes. After phagosome internalization into the deep cytoplasm, Syk activates PLCγ2 and hydrolyze PtdIns(4,5)P_2_, and its decrease leads to actin de-polymerization. Consequently, Syk might promote actin de-polymerization process via activation of PLCγ2. A previous report showed that phagosome maturation resulting from fusion with lysosome was delayed by transient assembly of F-actin^26^. More recently, it was showed that WHAMM initiates autolysosome tubulation by promoting actin polymerization^27^. Lysosome dynamics might be affected by F-actin. Rho family proteins-related phagosome modulation of F-actin remodeling has also been reported and the involvement of Syk in this pathway might be suspected^28^. Our study further illustrates the critical role of Syk-derived signaling that accelerates phagosome**-**lysosome fusion by regulating actin dynamics surrounding phagosomes (Fig. 7).

In the current study, we addressed the role of Syk in phagosome-lysosome fusion in complement-mediated phagocytosis and found that Syk-derived signaling interrupts the reconstruction of F-actin structure around phagosomes and accelerates phagosome-lysosome fusion. Since phagosome-lysosome fusion determines the fate of phagocytozed *C. albicans*, further studies of this process via Syk will be warranted for host defense against pathogens.

## Methods

### Reagents and antibodies

Fluorescein isothiocyanate (FITC) (Cat No. Z-2841), Texas Red (Cat No. Z-2843)-labeled zymosan, and Alexa Fluor 594-labeled *S. aureus* (Cat No. Z-23372) were purchased from Molecular Probes (Eugene, OR). FITC-labeled dextran 70 kDa (Cat No. 46945) was purchased from Sigma-Aldrich (St. Louis, MO). Anti-Syk monoclonal antibody (SYK-01) antibody (Cat No. sc-51703) and anti-integrin αM (2LPM19c) FITC monoclonal antibody (Cat No. sc-20050) were purchased from Santa Cruz Biotechnology (Santa Cruz, CA). Hoechst 33342 (Cat No. H342) was purchased from Dojindo (Kumamoto, Japan). LysoTracker Green DND-26 (Cat No. L7526), Alexa Fluor 594 phalloidin (Cat No. A12381), Alexa Fluor 488-conjugated goat anti-mouse IgG polyclonal antibody (Cat No. A32723), and Alexa Fluor 488-conjugated goat anti-rabbit IgG polyclonal antibody (Cat No. A11008) were purchased from Thermo Fisher Scientific (Waltham, MA). SiR-Actin kit (Cat No. CY-SC001) was purchased from Cytoskeleton, Inc. (Denver, CO). Anti-α-tubulin monoclonal antibody (Cat No. T9026, clone DM1A), and anti-acetylated tubulin monoclonal antibody (Cat No. T6793, clone 6-11B-1) were purchased from Sigma-Aldrich (St. Louis, MO). Anti-phospho-Syk (Tyr525/526) monoclonal antibody (Cat No. 2710, clone C87C1), anti-phospholipase C (PLC) γ2 monoclonal antibody (Cat No. 55512, clone E5U4T), anti-vinculin monoclonal antibody (Cat No. 13901, clone E1E9V), anti-cofilin monoclonal antibody (Cat No. 5175, clone D3F9) and anti-β-actin monoclonal antibody (Cat No. 8457, clone D6A8) were purchased from Cell Signaling Technology (Danvers, MA). RPMI1640 was purchased from Wako Pure Chemical Industries Ltd (Osaka, Japan, Cat No. 189-02025). Vitamin D_3_ was from Merck Millipore (Billerica, MA, Cat No. 679101). Phorbol 12-myristate 13-acetate (12-*O*-tetradecanoylphorbol-13-acetate, TPA, Cat No. P8139) was from Sigma-Aldrich (St. Louis, MO). Horseradish peroxidase (HRP) conjugated goat anti-rabbit (Cat No. 1706515) or goat anti-mouse (Cat No. 1706516) IgG (H + L) antibodies were from Bio-Rad (Hercules, CA). Jasplakinolide was from Merck Millipore (Billerica, MA, Cat No. 420107). U-73122 was from Cayman Chemical (Ann Arbor, MI, Cat No. 70740).

### Cell preparation and differentiation-induction

A human leukemic cell line HL60 was maintained in the culture medium (RPMI1640 medium containing 8% heat-inactivated fetal calf serum (FCS), 100 U/ml penicillin and 100 µg/ml streptomycin) in 5% carbon dioxide (CO_2_) humidified air at 37 °C. HL60 cells were induced to undergo differentiation to macrophage-like cells on 24-well plates (1 × 10^5^ cells/500 µl/well) or 3-well glass-base dish (2.5 or 5 × 10^4^ cells/200 µl/well) in culture medium containing 10^−7^ M vitamin D_3_ and 10 ng/ml TPA for 3 days.

Human peripheral blood mononuclear cells (PBMCs) of healthy volunteers were isolated by density-gradient centrifugation using Ficoll-Paque (Pharmacia Biotech AB, Uppsala, Sweden), and CD14^+^ monocytes were obtained using the magnetic cell sorting (MACS) system and microbeads conjugated with monoclonal mouse anti-human CD14 antibodies (Cat No. 130-050-201) purchased from Miltenyi Biotec (Bergisch Gladbach, Germany). Isolated monocytes were differentiated into macrophages on 3-well glass-base dishes at a density of 1 × 10^5^ cells/200 μl/well in culture medium containing 1 × 10^−7^ M vitamin D_3_ for 3 days.

### Cell surface expression of CR3

Macrophage-like differentiated HL60 cells on 24-well plates (1 × 10^5^ cells/500 μl/well) were scraped from the plates, incubated in 50 μl of PBS containing 2 μg/ml anti-integrin αM FITC antibody on ice for 1 h, and analyzed by flow cytometry (FACSCalibur; Becton Dickinson, SanJose, CA).

### Establishment of Syk-knockout (KO) HL60 cells

To establish HL60 cell line with Syk-knockout (KO) using CRISPR-Cas9-mediated gene disruption, two single-guide RNA (sgRNA) targeting *syk* gene were designed. These sgRNAs introduced deletion of nucleotides that causes a frameshift and premature stop codon within the open reading frame of the *syk* gene. These gRNAs were synthesized using the GeneArt Precision gRNA Synthesis Kit (Invitrogen, Waltham, MA, Cat. No. A29377). A mixture of sgRNA (240 ng) and Cas9 protein (1 μg) was transfected into 2 × 10^6^ HL60 cells using Neon Transfection system (Life technologies, Grand Island, NY). Single cell-derived Syk-KO cell clones were isolated by limiting dilution in 96-well plates. Syk-KO was verified by immunoblotting analysis of each clone.

### Immunoblotting analyses

HL60 cells were lysed with lysis buffer (62.5 mM Tris–HCl, pH 6.8; 2% SDS; 5% glycerol, 5% 2ME, BPB) and the lysates were separated in SDS-PAGE and transferred to PVDF membranes (Merck Millipore, MA, Cat No. IPVH00010). The membrane was blocked with 5% skim milk in T-TBS (10 mM Tris–HCl, pH 7.5; 100 mM NaCl; 0.1% Tween 20) and then incubated with corresponding antibodies in T-TBS for 45 min at room temperature (RT) or overnight at 4 °C. After incubation with HRP conjugated goat anti-rabbit or goat anti-mouse IgG antibodies for 30 min at RT, specific proteins were detected using an enhanced chemiluminescence immunoblotting system and LAS-3000 Lumino-Image Analyzer (Fuji Film; Tokyo, Japan).

### Preparation of *C. albicans*

*C. albicans* (NBRC 1594 strain) was obtained from Biological Resource Center of National Institute of Technology and Evaluation (Tokyo, Japan). *C. albicans* yeasts were incubated overnight at 37 °C on Sabouraud dextrose agar plate (Nissui Pharmaceutical Co. Ltd., Tokyo, Japan). Heat-killed *C. albicans* yeasts were obtained by water-bath treatment at 60 °C for 30 min. Killing of yeasts were confirmed by the absence of colonies on Sabouraud dextrose agar.

For FCS-induced hyphal formation, *C. albicans* yeasts (2 × 10^5^) were suspended in 50 mM citrate buffer (pH 5.5) or 50 mM phosphate buffer (pH 6.5) containing 10% FCS and incubated for 3 h at 37 °C in 3-well glass-base dish. After incubation in each pH condition, the yeasts were fixed in 4% formalin at 4 °C overnight and DIC images were acquired with a laser-scanning confocal microscope (LSM510 META; Carl Zeiss, Oberkochen, Germany).

### Opsonization with complement system

Zymosan particles (7.5 × 10^7^ particles/ml), *S. aureus* particles (1 × 10^9^ particles/ml), *C. albicans* yeasts (1.25 × 10^8^ yeasts/ml) were incubated in 50% human serum obtained from a healthy volunteer in PBS at for 30 min at 37 °C.

### Phagocytosis of *C. albicans* by macrophage-like differentiated HL60 cells

Macrophage-like differentiated HL60 cells on 3-well glass-base dishes (5 × 10^4^ cells/200 μl/well) were incubated with serum-opsonized *C. albicans* yeasts (1 × 10^5^) for 15 min at 37 °C. After removing unbound yeasts, the cells were further incubated for 8 h at 37 °C and fixed overnight in 4% formalin at 4 °C followed by washing with 0.05% Tween 20 in PBS (PBST). Then, extracellular *C. albicans* were stained with 5 μg/ml Calcofluor White in PBST for 10 min at RT. Widefield-microscopy images were acquired with a laser-scanning confocal microscope.

### Measurement of phagosomal pH

Macrophage-like differentiated HL60 cells on 24-well plates (1 × 10^5^ cells/500 μl/well) were incubated with 5 × 10^5^ particles/50 μl/well serum-opsonized FITC- or Texas Red-labeled zymosan for 15 min at 37 °C. After removing unbound zymosan completely, the cells were incubated for indicated chase intervals (15, 60, 120 and 300 min) at 37 °C. At the designated time points, the cells were scraped from the plates and analyzed by flow cytometry. Phagosomal pH of the cells was determined by calculating the mean FITC/Texas Red fluorescence ratio and extrapolating from the calibration curve. The calibration curve data were obtained by suspending the FITC- or Texas Red-labeled zymosan particles in citrate or phosphate buffers at a fixed pH (ranging from pH 3.5 to 8) and by plotting the mean FITC/Texas Red fluorescence ratio for each zymosan particles versus pH.

### Analysis of FITC-labeled dextran 70 kDa uptake

Macrophage-like differentiated HL60 cells on 24-well plates (1 × 10^5^ cells/500 μl/well) were incubated with 20 μl/well of FITC-labeled dextran 70 kDa (6.5 mg/ml) for 30 min at 37 °C. After removing unloaded dextran, the cells were scraped from the plates and analyzed by flow cytometry.

### Immunofluorescence staining of the cells

Macrophage-like differentiated HL60 cells on 3-well glass-base dishes (2.5 × 10^4^ cells/200 μl/well) were fixed with 4% formalin for 1 h, permeabilized with 0.5% Triton X-100 in PBS for 5 min, and incubated overnight with mouse anti-α-tubulin (1:500 dilution), mouse anti-acetylated tubulin (1:400 dilution), rabbit anti-phospho-Syk (Tyr525/526) (1:200 dilution), or rabbit anti-PLCγ2 (1:200 dilution) monoclonal antibodies, followed by incubation with 1:100 dilution of Alexa Fluor 488-conjugated goat anti-mouse or anti-rabbit IgG polyclonal antibodies and 1 μg/ml Hoechst 33342 for 30 min. Actin staining was performed similarly with Alexa Fluor 594-conjugated phalloidin (1:33 dilution) in PBST for 30 min at RT. Fluorescence-stained cells were observed with a laser-scanning confocal microscope.

### Analysis of LysoTracker localization in phagosomes

Macrophage-like differentiated HL60 cells on 3-well glass-base dishes (2.5 × 10^4^ cells/200 μl/well) were preincubated with 2 μM LysoTracker Green for 15 min, and further incubated with serum-opsonized Texas Red-labeled zymosan (1 × 10^6^ particles/well) for 15 min at 37 °C. After removing unbound zymosan, the cells were incubated in the presence of LysoTracker Green for indicated chase intervals at 37 °C, and microscopic images were acquired with a laser-scanning confocal microscope. For live cell imaging, Incubator PMS (PeCon GmbH, Erbach, Germany) was mounted on the microscope stage and Temperature Module S1 and CO_2_ Module S1 were used to maintain the cells at culture condition. For three-dimensional (3D) live cell imaging, a series of Z-stacks were recorded at 0.54 μm intervals, and the images were recorded at 31.43 s intervals for time-lapse live cell imaging.

### Live cell imaging of F-actin and lysosomes

Macrophage-like differentiated HL60 cells or monocytes on 3-well glass-base dishes were preincubated with 100 nM SiR-actin and 2 μM LysoTracker Green for 2 h and 15 min, respectively, and further incubated with serum-opsonized heat-killed *C. albicans* yeasts (1 × 10^5^ yeasts/well) for 15 min at 37 °C. After removing unbound yeasts, the cells were incubated in the presence of SiR-actin and LysoTracker Green for 2 h at 37 °C, and confocal fluorescence and DIC images of living cells were acquired after 2 h chase.

### Electron microscopy

Macrophage-like differentiated HL60 cells on 3-well glass-base dishes were incubated with serum-opsonized FITC-labeled zymosan as described above for 2 h. The cells were fixed with 2% paraformaldehyde/2.5% glutaraldehyde/1% tannic acid in PBS, followed by post-fixation in 2% osmium tetroxide, ethanol dehydration and embedding in Epon resin. Thin sections by ultramicrotomy were observed with a transmission electron microscopy.

### Statistical analysis

In some experiments, statistical significance was determined by the Student’s two-tailed *t-*test and one-way analysis of variance (ANOVA).

### Ethics approval

This study included the experiments using human subjects. Therefore, this study was approved by the ethics committee of Himeji Dokkyo University based on the Declaration of Helsinki and the ethical guidelines for medical and health research involving human subjects by Ministry of Education, Culture, Sports, Science and Technology of Japan. Primary monocytes and serum were obtained from the peripheral blood of healthy volunteers after informed consent.

## Supplementary Information

Supplementary Information.

Supplemental Video 1

Supplemental Video 2

Supplemental Video 3

Supplemental Video 4
