# Supplementary material for: Syk facilitates phagosome-lysosome fusion by regulating actin-remodeling in complement-mediated phagocytosis
Source: Sci Rep. 2020 Dec 16;10:22086. doi: 10.1038/s41598-020-79156-7 (PMC7744523; doi:10.1038/s41598-020-79156-7)
Supplement: Supplementary file 1 — Supplementary Information. [file 41598_2020_79156_MOESM1_ESM.pdf]

Syk facilitates phagosome-lysosome fusion by regulating actin-remodeling in complement- mediated phagocytosis

Hiroyuki Tabata[1], Hiroyuki Morita[1], Hiroaki Kaji[1], Kaoru Tohyama[2], and Yumi Tohyama[1]*

[1]Division of Biochemistry, Faculty of Pharmaceutical Sciences, Himeji Dokkyo University, Hyogo 670-8524, Japan;
[2]Department of Laboratory Medicine, Kawasaki Medical School, Okayama 701-0192, Japan

***Address correspondence to**:
Yumi Tohyama,
Division of Biochemistry, Faculty of Pharmaceutical Sciences, Himeji Dokkyo University, 7-2-1 Kami-ohno, Himeji, Hyogo 670-8524, Japan
Tel.: 81-79-223-6809; Fax: 81-79-285-0352;
E-mail: ytohyama@gm.himeji-du.ac.jp

Supplementary Figures and Legends

a

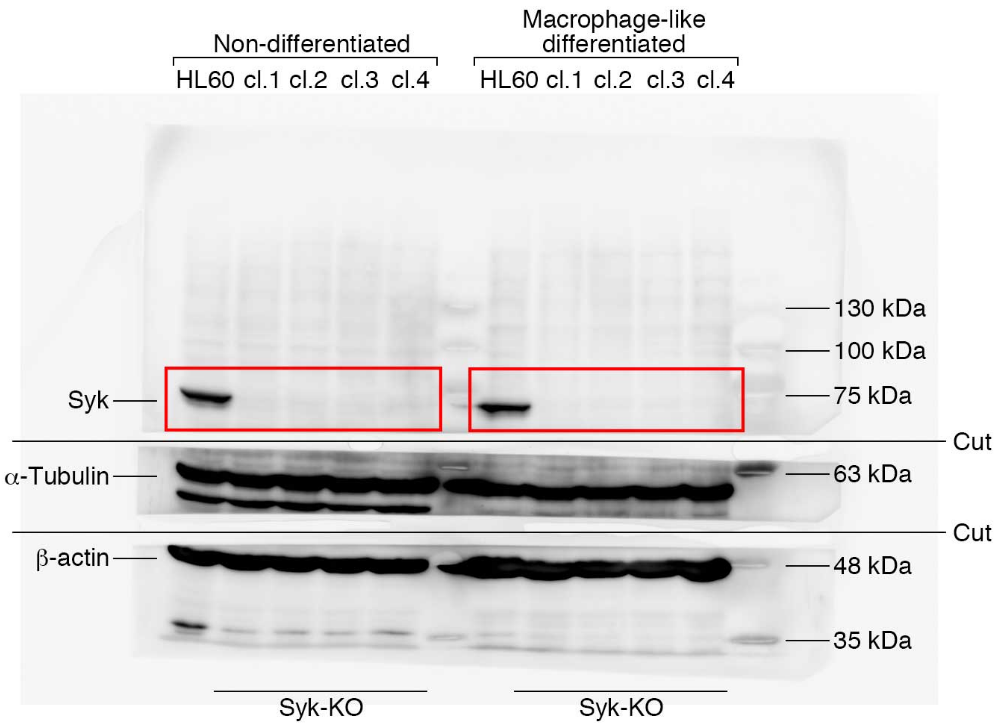

b

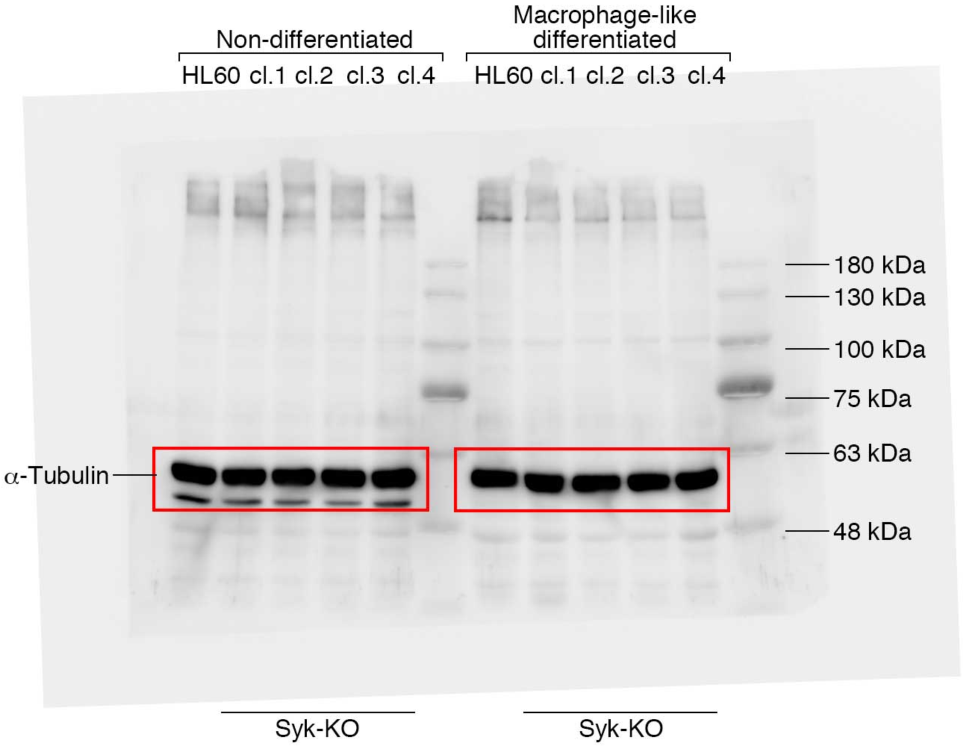

Figure S1. The original images of the immunoblotting analysis in Figure 1a.

The same samples were loaded on both upper (a) gel (20 µg per lane) and lower (b) gel (7.5 µg per lane). In (a), the blotted membrane was divided into three sections and they were treated with different antibodies as indicated. The areas shown in Figure 1a were indicated by red frames of the original gels.

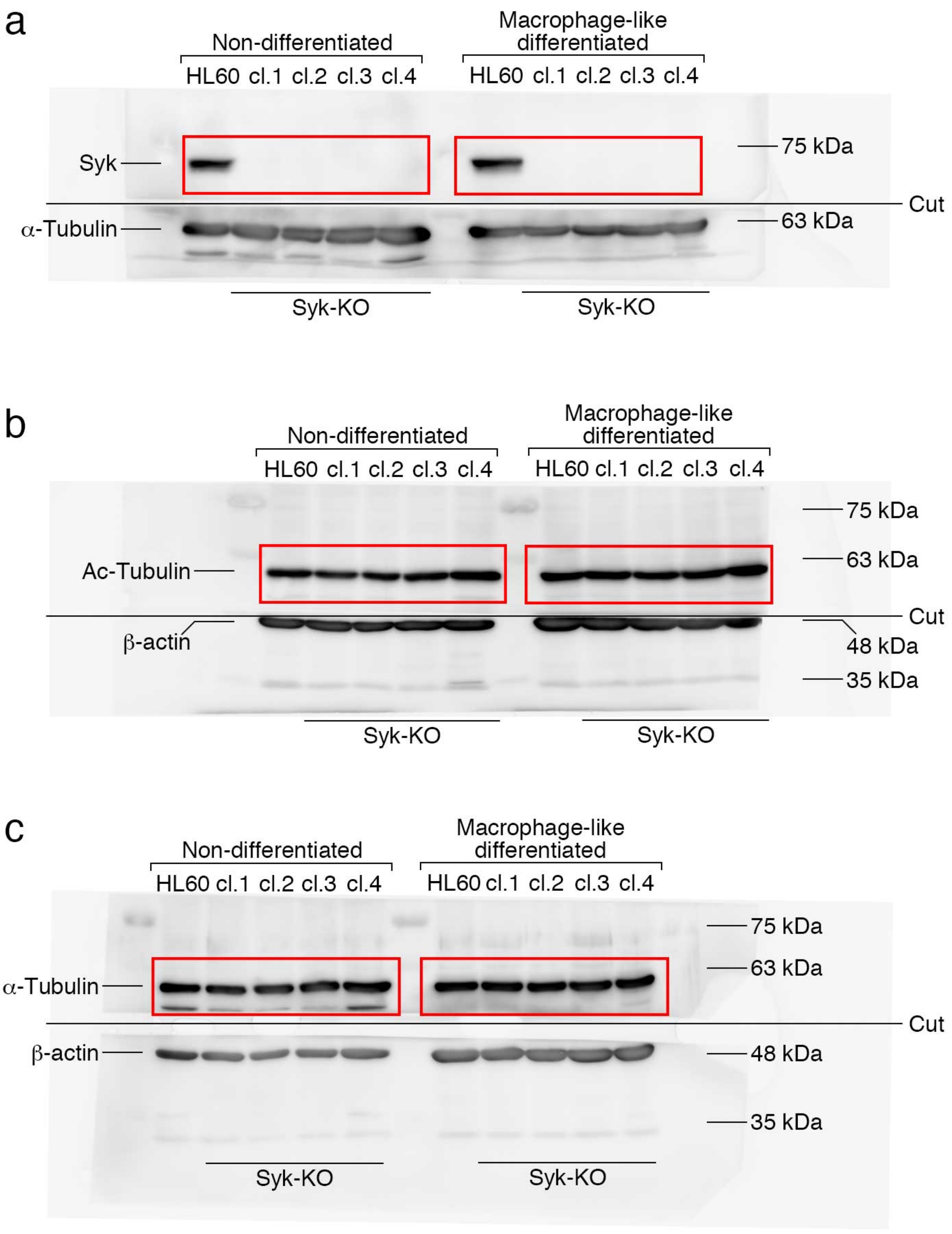

Figure S2. The original images of the immunoblotting analysis in Figure 3c.

The same samples were loaded on upper (a) (20 µg per lane), middle (b) (20 µg per lane) and lower (c) (7.5 µg per lane) gels. Each blotted membrane was divided into two sections and they were treated with different antibodies as indicated. The areas shown in Figure 3c were indicated by red frames of the original gels.

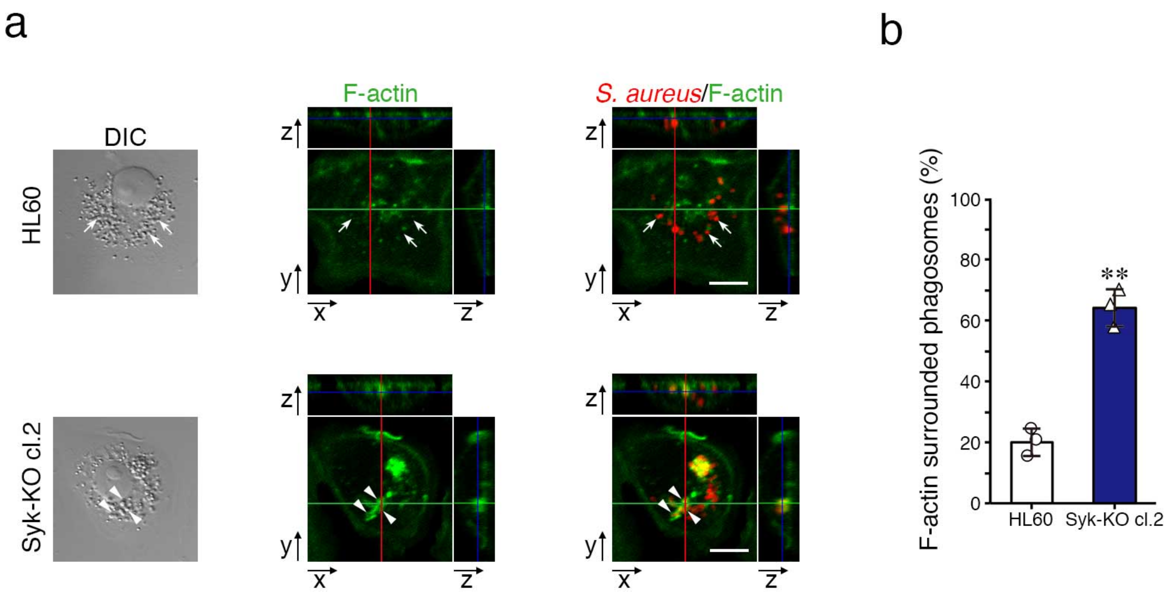

Figure S3. a Representative images of F-actin (FITC-labeled phalloidin) during phagocytosis of macrophage-like differentiated parental and Syk-KO HL60 cells after incubation with complement-opsonized Alexa Fluor 594-*S. aureus* particles for 15 min, followed by incubation for 2 h, as quantified in (b). Arrows show the phagosomes that are not surrounded by F-actin and arrowheads show the phagosomes surrounded by F-actin. Scale bars show 10 µm. b Quantification of the percentage of the phagosomes surrounded by F-actin in macrophage-like differentiated parental and Syk-KO HL60 cells during phagocytic process. More than 100 phagosomes per single clone were analyzed. Data show the means ± SD derived from three independent experiments. p-values were calculated using a two-tailed unpaired Student's *t*-test. ** < 0.01.

a

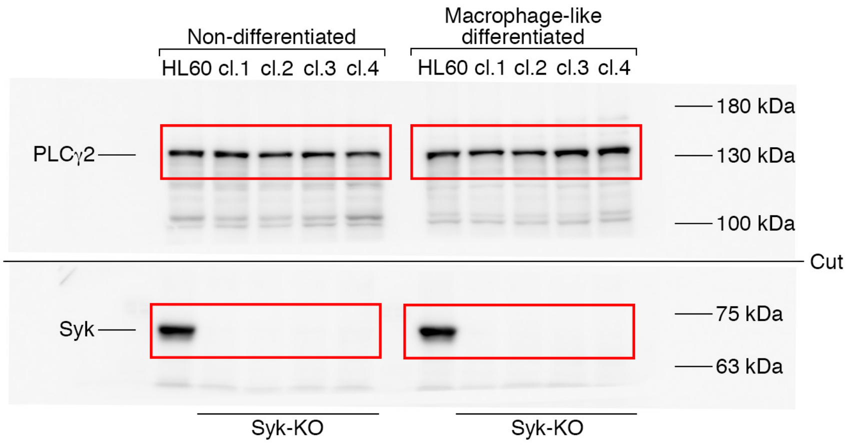

Non-differentiated
Macrophage-like differentiated
HL60 cl.1 cl.2 cl.3 cl.4
HL60 cl.1 cl.2 cl.3 cl.4
PLCγ2
180 kDa
130 kDa
100 kDa
Cut
Syk
75 kDa
63 kDa
Syk-KO
Syk-KO

b

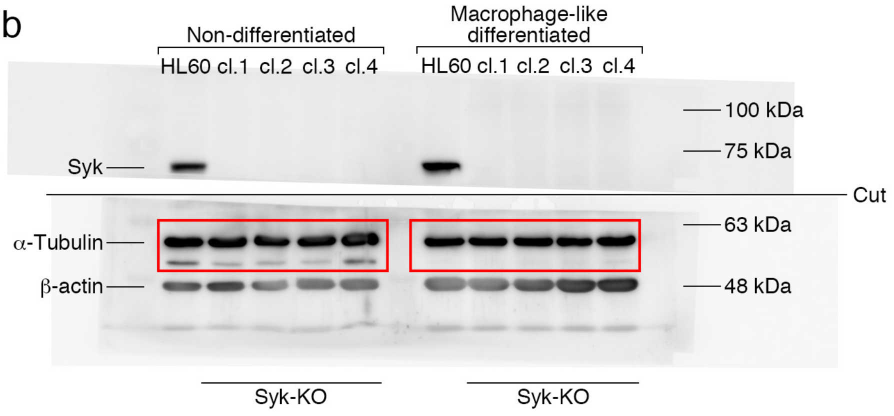

Non-differentiated
Macrophage-like differentiated
HL60 cl.1 cl.2 cl.3 cl.4
HL60 cl.1 cl.2 cl.3 cl.4
Syk
100 kDa
75 kDa
Cut
α-Tubulin
β-actin
63 kDa
48 kDa
Syk-KO
Syk-KO

c

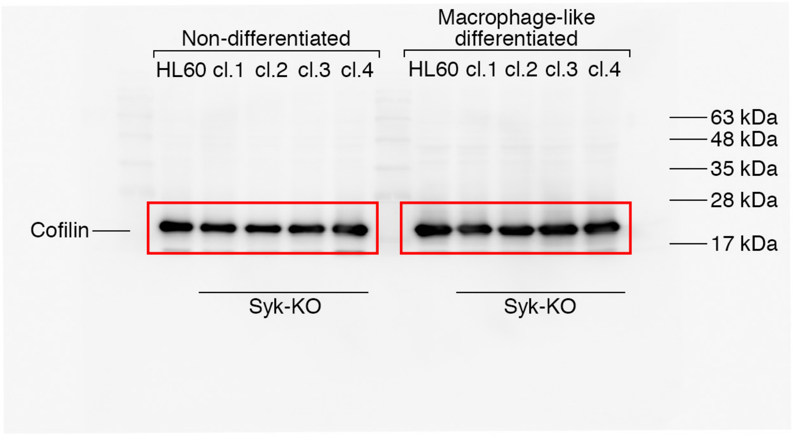

d

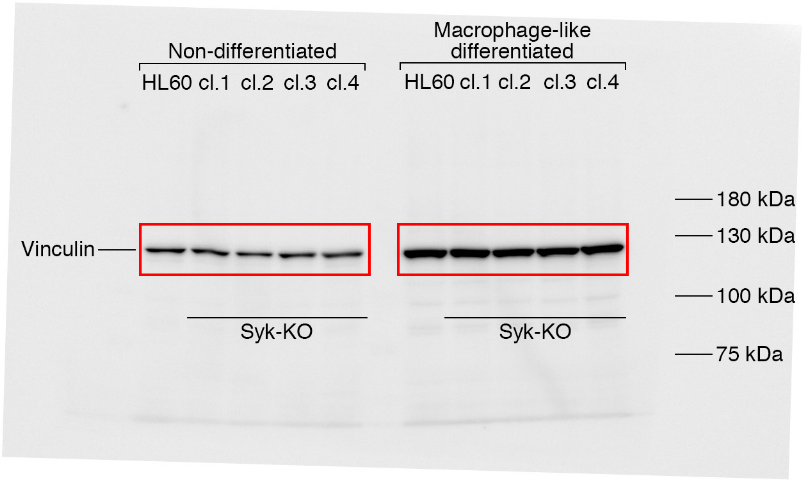

Figure S4. The original images of the immunoblotting analysis in Figure 6b.

The same samples were loaded on (a) (15 µg per lane), (b) (7.5 µg per lane), (c) (15 µg per lane) and (d) (7.5 µg per lane) gels. In (a) and (b), blotted membranes were divided into two sections and they were treated with different antibodies as indicated. In (c) and (d), blotted membranes were treated with anti-Cofilin or anti-Vinculin antibody, respectively. The areas shown in Figure 6b were indicated by red frames of the original gels.

a

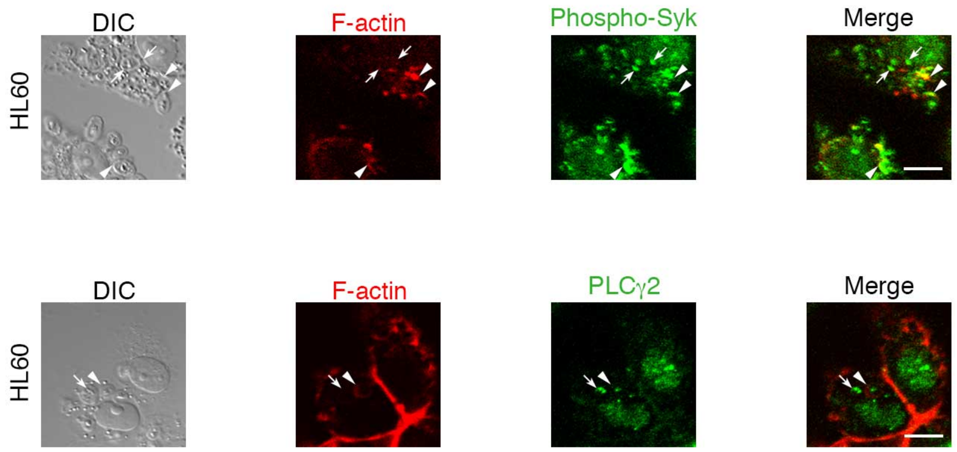

Figure S5. Representative images of immunofluorescence staining using antibodies against phospho-Syk (Tyr525/526) (upper panel) or PLCγ2 (lower panel), during phagocytosis of macrophage-like differentiated parental HL60 cells after incubation with complement-opsonized zymosan particles for 1 h. Each sample was co-stained with Alexa Fluor 594-labeled phalloidin for F-actin. Arrows show the phagosomes that are not surrounded by F-actin and arrowheads show the phagosomes surrounded by F-actin. Scale bars show 10 µm.

Legends of Supplemental Videos

Supplemental Video 1 and 2: Phagosome-lysosome fusion during phagocytosis in macrophage-like differentiated parent and Syk-KO HL60 cells.

Parental (Supplemental Video 1) and Syk-KO (Supplemental Video 2) macrophage-like differentiated HL60 cells were pulsed with complement-opsonized Texas Red-zymosan for 15 min and chased in culture medium containing 1 μM LysoTracker Green (green). DIC and fluorescence images were taken from 35 min to 75 min after addition of zymosan particles every 30 sec. Video speed is 2 frames/second. Scale bars, 10 μm.

Supplemental Video 3 and 4: Periphagosomal F-actin assembly and phagosomal acidification during phagocytosis in macrophage-like differentiated parent and Syk-KO HL60 cells.

Parental (Supplemental Video 3) and Syk-KO (Supplemental Video 4) macrophage-like differentiated HL60 cells were pulsed with complement-opsonized-heat killed *C. albicans* yeasts for 15 min and chased in culture medium containing 100 nM SiR-actin (magenta) and 1 μM LysoTracker Green (green). DIC and fluorescence images were taken from 15 min to 57 min after addition of *C. albicans* yeasts every 60 sec. Video speed is 2 frames/second. Scale bars, 5 μm.
